# What do women undergoing *in vitro* fertilization (IVF) understand about their chance of IVF success?

**DOI:** 10.1093/humrep/dead239

**Published:** 2023-11-17

**Authors:** C McMahon, K Hammarberg, S Lensen, R Wang, B W Mol, B J N Vollenhoven

**Affiliations:** Department of Obstetrics & Gynaecology, Monash University, Melbourne, VIC, Australia; School of Public Health and Preventative Medicine, Monash University, Melbourne, VIC, Australia; Victorian Assisted Reproductive Treatment Authority (VARTA), Melbourne, VIC, Australia; Department of Obstetrics and Gynaecology, Royal Women’s Hospital, University of Melbourne, Melbourne, VIC, Australia; Department of Obstetrics & Gynaecology, Monash University, Melbourne, VIC, Australia; Department of Obstetrics & Gynaecology, Monash University, Melbourne, VIC, Australia; Aberdeen Centre for Women’s Health Research, Institute of Applied Health Sciences, School of Medicine, Medical Sciences and Nutrition, University of Aberdeen, Aberdeen, United Kingdom; Women’s and Newborn Program, Monash Health, Melbourne, VIC, Australia; Department of Obstetrics & Gynaecology, Monash University, Melbourne, VIC, Australia; Women’s and Newborn Program, Monash Health, Melbourne, VIC, Australia; Monash IVF, Melbourne, VIC, Australia

**Keywords:** IVF, fertility, chance, success, understanding, knowledge, information, explanation, estimate

## Abstract

**STUDY QUESTION:**

How well informed are Australian women who undergo IVF about their chances of having a baby?

**SUMMARY ANSWER:**

Only one in four women estimated their individual chance of success with IVF accurately, with most women overestimating their chance.

**WHAT IS KNOWN ALREADY:**

Limited knowledge about infertility and infertility treatment in the general population is well-documented. The few studies that have investigated patients’ knowledge about the chance of IVF success suggest that while IVF patients are aware of average success rates, they tend to be unrealistic about their own chance of success.

**STUDY DESIGN, SIZE, DURATION:**

We conducted an anonymous online survey of 217 women who had started IVF since 2018 in Australia. The survey was advertised on social media, enabling women from across Australia to participate. Responses were collected in June 2021.

**PARTICIPANTS/MATERIALS, SETTING, METHODS:**

The survey included questions on demographic characteristics and IVF history. It asked what participants thought their chance of having a baby from one IVF treatment cycle was, how they rated their knowledge about chance of success, and about their experience of receiving IVF-related information. Participants’ estimations of their chance of success were compared with their chance as calculated by the Society for Assisted Reproductive Technology’s (SART) online calculator. Responses to a free-text question about what information women wished they had been given when they started treatment were analysed thematically.

**MAIN RESULTS AND THE ROLE OF CHANCE:**

Only about a quarter (58/217, 27%) of participants accurately estimated their chance of having a baby within 20% relative to their SART calculated chance, with more than half (118/217, 54%) overestimating their chance. Ninety percent of women indicated that their preferred source of treatment information was a consultation with their doctor, despite less than half (44%) reporting that doctors explained the probability of having a baby with IVF well (mean 5.9/10). In free-text responses, many women also reported that they wished they had been given more realistic information about IVF and their chance of success.

**LIMITATIONS, REASONS FOR CAUTION:**

The dissemination method precludes calculation of response rate, and it is not possible to know if participants are representative of all women undergoing IVF. Additionally, we only surveyed women undergoing IVF, while those who decided not to have IVF were not included. Therefore, women who overestimated their chance may have been overrepresented. There is also inherent imprecision in the way understanding of chance of success was estimated. The potential impact of recall bias could neither be quantified nor excluded. It is difficult to determine to what extent women’s lack of understanding of what is possible with IVF is due to poor information-provision by clinicians and the clinic, and how much can be explained by optimism bias.

**WIDER IMPLICATIONS OF THE FINDINGS:**

The finding of poor understanding of personal chance of success amongst women undergoing IVF in Australia requires further investigation to determine potential reasons for this. The findings can be used by clinics to develop strategies for improvement in the information-provision process to ensure that women can make informed decisions about their fertility treatment.

**STUDY FUNDING/COMPETING INTEREST(S):**

This study received no external funding. S.L. is supported by a NHMRC Investigator Grant (APP1195189). R.W. is supported by a NHMRC Investigator Grant (APP2009767). B.W.M. is supported by a NHMRC Investigator Grant (GNT1176437). B.W.M. reports consultancy for Merck and ObsEva and has received research funding and travel funding from Merck. The other authors have no conflicts of interest.

**TRIAL REGISTRATION NUMBER:**

N/A.

## Introduction

Despite the increasing use of IVF and related assisted reproductive technologies worldwide, success rates are still modest, with only around 25–30% of cycles resulting in a live birth (de Mouzon *et al.*, 2020). As such, it is important that couples undergoing IVF are well informed about their chances of success prior to starting treatment.

Strong evidence exists for a lack of knowledge about fertility and treatments for infertility in the general population (Hammarberg *et al.*, 2013; Maeda *et al.*, 2015; Kudesia *et al.*, 2017; Pedro *et al.*, 2018; Cheung *et al.*, 2019). There is a suggestion that this is less of a problem among women undergoing IVF, as they appear to be aware of approximate success rates (Maheshwari *et al.*, 2008). However, some studies have concluded that despite women knowing about average success rates, they expect their own chance of success to be above average (Miron-Shatz *et al.*, 2021). In one study exploring the expectations of IVF patients, researchers found that many patients had unrealistically high expectations of success (Devroe *et al.*, 2022). Their study cited ‘dispositional optimism’ as a leading contributor to patients’ expectations.

Because about half of patients who undergo fertility treatment do not achieve a live birth, it has been argued that looking at ‘process indicators’, such as patient-centredness, alongside treatment outcomes is crucial (Dancet *et al.*, 2011; Gonen, 2016). Patient-centred care refers to care that is ‘responsive to individual patient needs and guided by patient values’ (Dancet *et al.*, 2010; van Empel *et al.*, 2011), rather than purely the clinician’s evaluation of the patient’s physical condition (Dancet *et al.*, 2014). In a Dutch study where researchers designed and validated a patient-centredness questionnaire in a large population of fertility treatment patients, patients rated factors related to ‘information’ as very important but reported that this was often insufficiently demonstrated by fertility clinics (van Empel *et al.*, 2010). Of all the aspects of care they considered, patients placed the highest level of importance on being provided with an honest and clear idea of what to expect from their treatment. What is not known is whether patients feel these needs are being adequately met, and whether current information provision results in patients having a good understanding of their treatment.

The gap in the existing literature is that, to the best of our knowledge, there is no evaluation of the accuracy of women’s understanding of their own chance of success, by comparing what they estimate their chance of success to be to a more objective prediction. The purpose of this study is to evaluate women’s understanding of their IVF treatment, particularly their personal chance of live birth, and to identify whether their information needs are met. We hypothesized that women would overestimate their personal chance of success with IVF.

## Materials and methods

### Eligibility and recruitment

This was an online survey of women living in Australia who had started IVF treatment since 2018 (Supplementary Data File S1).

The survey was advertised on Facebook and Instagram in a targeted advertising campaign from 3 to 21 June 2021. The advertisement was displayed to women living in Australia who had shown interest in IVF or infertility through the pages and groups they associated with on Facebook.

### Survey design

The survey software Qualtrics (Qualtrics, 2021) was used to administer the questionnaire which was composed of a combination of multiple choice, free-text response, and slider questions and took ∼10 min to complete. Using Dancet *et al.*’s framework for patient-centred care (Dancet *et al.*, 2014), the study-specific questionnaire was developed with the input of several researchers with expertise in IVF research and questionnaire design. The questionnaire was structured around two main aspects of understanding: women’s knowledge of their personal chance of IVF success and the Choosing Wisely questions, a set of questions designed to empower patients to ask their healthcare provider about risks, alternative options and costs, before proceeding with a proposed treatment (Choosing Wisely Australia, 2021). The questionnaire also included demographic questions and questions about the causes of infertility, to match the inputs required for the Society for Assisted Reproductive Technology (SART) Online Calculator.

Five women from the University of Melbourne’s (In)fertility Research Panel (The University of Melbourne, 2021), who fit the study’s eligibility criteria, piloted the questionnaire and provided feedback, which resulted in minor modifications to the survey.

The SART Online Calculator was developed to help women understand their chance of success and is based on the data from over 320 000 women in the USA (Society for Assisted Reproductive Technology, 2021). It uses data such as age, height, and weight (to calculate BMI) and infertility diagnosis to calculate a predicted chance of IVF success. In our study, participants’ demographic and fertility-related information was entered into the SART Online Calculator to determine their calculator-predicted chance of a live birth after one cycle of IVF. The calculated chance was then compared to the participant’s response to the question that asked: ‘At the time of your first IVF cycle, roughly what did you think your chance of having a baby was, after one complete IVF treatment cycle?’. The percentage comparison value was calculated as the difference between the participant-predicted and the calculator-predicted chance, divided by the calculator-predicted chance. For example, a participant with a calculator-predicted chance of 20% who estimated their chance to be 25% has a percentage comparison of 25% ((25–20)/20 = 25%). Participants were considered to have accurately estimated their chance of success if their percentage comparison value was 20% or less.

### Statistical analysis

The quantitative data were analysed in SPSS Statistics 27 (IBM Corp, 2020) and Stata 18.0 (StataCorp, 2023). Descriptive statistics were used to describe characteristics of participants. The associations between the following characteristics and the participants’ expectation of live birth were evaluated in multinomial logistic regressions: participants’ rating of their understanding and participants’ rating of their clinician’s explanation on the accuracy of estimation. The associations were expressed as odds ratios (ORs) (both unadjusted and adjusted) with 95% confidence intervals and age was considered as a confounding factor in the adjusted model. Only records where participants answered all mandatory questions (relating to their chance of success and demographic factors required for the success calculator) were included in the analysis. Where data were missing for certain questions, percentages were calculated with missing data treated as ‘no response’, and not included in the denominator.

NVivo 12 was used by one researcher (CM) to code free-text responses through inductive thematic analysis (QSR International Pty Ltd, 2018). Thematic analysis was selected over other qualitative methods as it is widely used, and therefore widely understood, has the flexibility to be adapted to the data and research question, and allows for a rich summary of the data. The codes that were created from the textual responses each represent an idea present in the data, relevant to the specific aims of the study. The codes were organized into themes in an iterative process, with themes representing patterns in the data. These themes were checked by two other members of the research team (B.J.N.V. and K.H.) who had not been involved in the coding process. Bubble charts were created to illustrate the common ideas and to demonstrate the relative frequency of each idea in the data.

### Ethics approval

This study was conducted in compliance with the National Statement on Ethical Conduct in Human Research (The National Health and Medical Research Council (NHMRC), The Australian Research Council, Universities Australia, 2007 (Updated 2018)). Ethics approval for the survey was obtained from the Monash Health Human Research Ethics Committee (HREC) on 5 May 2021 (Reference Number: RES-21-0000-169L). Following ethics approval from Monash Health, the survey was registered with Monash University Human Research Ethics Committee (MUHREC) on 13 May 2021 (Project number: 28050).

## Results

A total of 518 women started the survey, of whom 217 completed the mandatory questions and were included in the analysis. There were no statistically significant differences in background information between participants who started the survey but did not complete it and those who completed the survey. The demographic characteristics of participants are summarized in Table 1. The mean participant age was 35 years and most participants (68%) had undergone one or two IVF cycles.

**Table 1.** Demographic characteristics of the participants.

	Participants (n = 217)
**Characteristic, mean (SD)**	
Age (years)	34.8 (4.6)
BMI (kg/m^2^)	28.2 (6.7)
**Highest level of completed education, n (%)**	
Secondary school	28 (12.9)
Certificate (including apprenticeship)	30 (13.8)
Diploma (including advanced diploma)	37 (17.1)
Bachelor’s Degree	78 (35.9)
Postgraduate qualifications (e.g. Masters, PhD)	43 (19.8)
**Type of fertility treatment, n (%)**	
IVF (including ICSI) only	179 (82.5)
IVF (including ICSI) and IUI	38 (17.5)
**Year commenced IVF/ICSI, n (%)**	
2018	28 (12.9)
2019	41 (18.9)
2020	86 (39.6)
2021	62 (28.6)
**Number of IVF/ICSI cycles, n (%)**	
1	90 (41.5)
2	60 (27.6)
3	27 (12.4)
4	40 (18.4)
**Number with pregnancies/live births from IVF/ICSI, n (%)**	
Pregnancies not resulting in live birth	40 (18.4)
Live births	70 (32.3)

Only a quarter (58/217, 27%) of participants predicted their chance of live birth within 20% relative to their calculator-predicted chance, whereas more than half (118/217, 54%) overestimated their chance. When asked to rate their understanding of their chance of having a baby from one cycle of IVF (on a scale of 1 to 10), the mean response was 6.6 (SD = 2.1), with more than half of women (119/217, 55%) rating their level of understanding as high (7-10/10). For every one score higher that participants rated their understanding, they were significantly less likely to overestimate their chance of success (adjusted OR 0.83, 95% CI: 0.71–0.98) (Table 2).

**Table 2.** Association between participant’s rating of their own understanding of their chance of IVF success and their rating of explanation quality with under/overestimation of chance of IVF success.

	Underestimation		Overestimation	
Characteristics	Unadjusted OR (95% CI)	* **Adjusted OR (95% CI)**	Unadjusted OR (95% CI)	* **Adjusted OR (95% CI)**
**Level of understanding**	1.11 (0.91–1.37)	1.11 (0.91–1.37)	0.84 (0.72–0.98)	0.83 (0.71–0.98)
**Quality of explanation**	0.96(0.78–1.09)	0.98 (0.84–1.13)	0.86 (0.76–0.96)	0.87 (0.77–0.98)

Accurate estimation was used as the reference in multinomial logistic regression.

* Female age was considered as a confounding factor (continuous variable).

**Figure 1.**
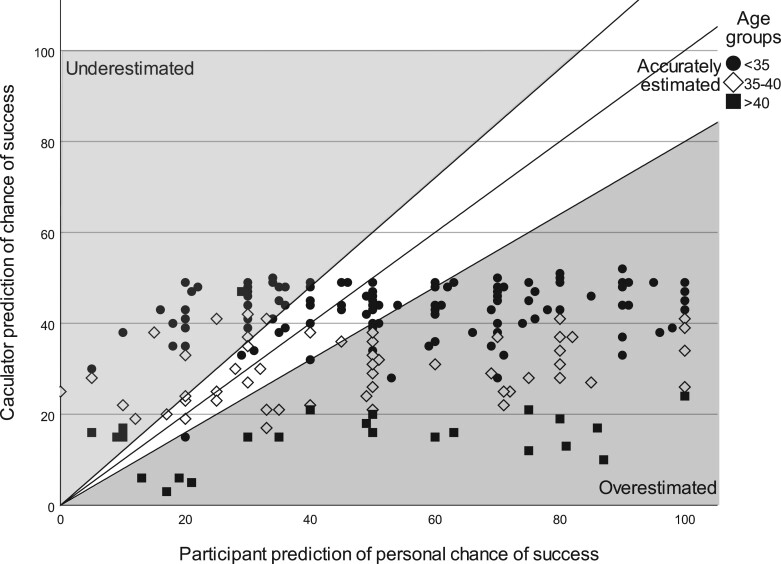
**Scatter plot of participant’s prediction of their personal chance of success compared to their SART calculator-predicted chance.** Participants differentiated by age group.

Figure 1 shows every participant’s prediction of their personal chance of success plotted against their calculator-predicted chance.

**Figure 2.**
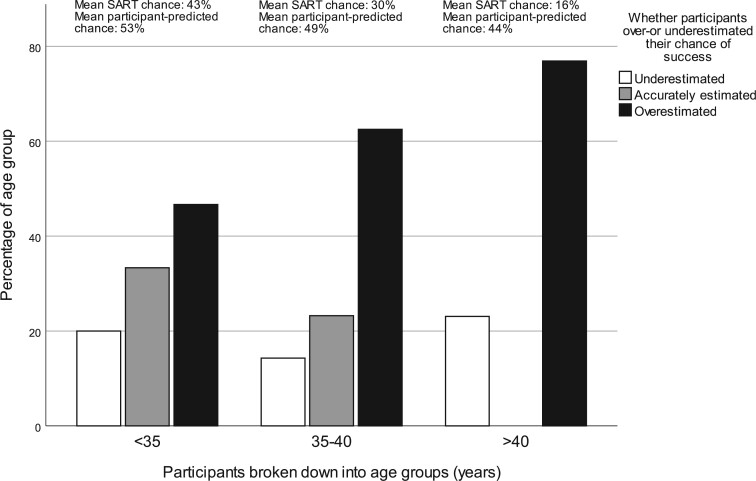
Participants grouped by age and whether they over- or underestimated their chance of success.

Every year of increase in participant age was associated with higher odds of both overestimation (OR 1.12, 95% CI 1.04–1.21) and underestimation (OR 1.11, 95% CI 1.01–1.22). Participants over the age of 40 were three times more likely to overestimate their chance of success than younger participants (OR 3.16, 95% CI 1.22–8.22) (Fig. 2).

**Figure 3.**
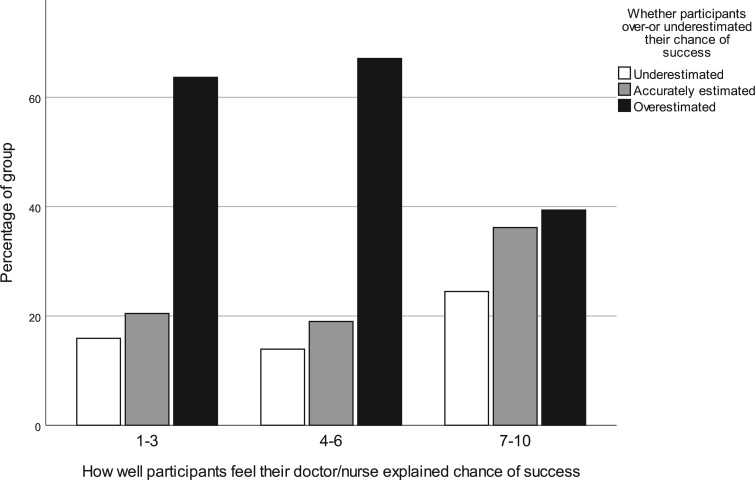
Participants grouped by how well they felt their doctor or nurse explained their chance of success, and whether they over- or underestimated their chance of success.

As can be seen in Fig. 3 and Table 2, participants who felt their doctor or nurse explained their chance of success better (for every one score higher in rating) were significantly less likely to overestimate their chance of success (OR 0.87, 95% CI 0.77–0.98).

Almost all participants (90%) indicated that a consultation with their doctor was one of their preferred ways to get information about their fertility treatment. A consultation with a nurse and searching the internet were also frequently chosen options, with just over half of participants selecting each of these (Table 3).

**Table 3.** Participants’ preferred source of information about their fertility treatment.

Preferred source of information about fertility or fertility treatment	Percentage of participants (%)
Consultation with doctor	90.3
Internet searching	54.8
Consultation with nurse	53.9
Written information, e.g. pamphlet	32.3
Social media	31.8
Talking to friends/family	27.6
Journal or research articles	24.4
Videos	21.7
Books	9.2

The mean ratings participants gave for the explanations they received from their doctor or nurse in four domains of information, as well as how well informed they felt overall, are shown in Table 4.

**Table 4.** Mean participant ratings (out of 10) of how well they felt their doctor or nurse explained various aspects of their treatment, and how well informed they felt overall.

Aspect of care	Participant rating (out of 10) Mean (SD)
Treatment process	6.9 (2.4)
Potential risks or side effects	5.6 (2.9)
Probability of having a baby from IVF	5.9 (2.9)
Probability of having a baby without any treatment	5.6 (3.6)
Overall, how well informed did you feel about your IVF treatment before you started?	6.2 (2.6)

**Figure 4.**
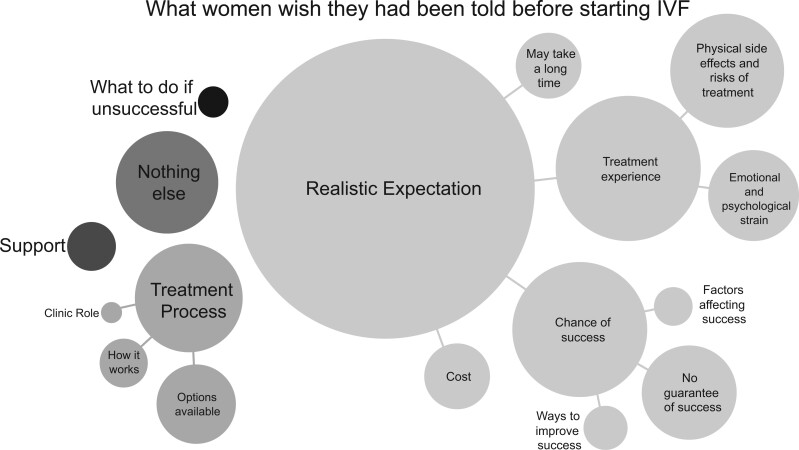
**Bubble chart summary of participants’ responses to what they wish they had been told before starting IVF**.

Figure 4 is a summary of participant responses to the free-text question ‘Is there anything you were not told before starting IVF/ICSI treatment that you wish you had been told?’. The area of each bubble in the figure is proportional to the number of times that idea was mentioned. Most of the responses related to the idea of having a realistic expectation, mainly through understanding their chance of success and what to expect from the experience of undergoing IVF treatment. The broader theme of ‘realistic expectation’ includes several sub-themes that were common in participant responses. For example, the ‘no guarantee of success’ bubble represents comments such as the following:

‘IVF does not guarantee a baby. Or a pregnancy’—*39-year-old woman who has had 2 cycles of IVF*

‘It [IVF] does not mean you are guaranteed first time or even second to third time around’—*30-year-old woman who has had 3 cycles of IVF*

Participants also wished they had known:

‘The potential effects of medications on the body’—*35-year-old woman who has had 5 cycles of IVF (Physical side effects of treatment sub-theme)*

‘What next steps were if a treatment didn’t work’—*33-year-old who has had 4 cycles of IVF (Options available sub-theme)*

‘The complete expense of treatments broken down. There have been a lot of hidden costs’—*32-year-old woman who has had 4 cycles of IVF (Cost sub-theme)*

The ‘Nothing else’ theme represents participants who stated that there was nothing else they wish they had been told.

## Discussion

This study found that, despite rating their understanding of chance of success as high, most women undergoing IVF overestimate their personal chance of having a baby after treatment. Concerningly, women with higher age were less likely to accurately estimate their chance of success than younger participants. It also revealed that women want their fertility clinic to provide realistic and personalized information about their chance of IVF success.

Our study confirms the findings of the study by Devroe *et al.* (2022) that women hold unrealistic expectations when undergoing IVF, but our comparison with estimates of chance from the SART calculator allows us to quantify this. The reasons for women overestimating their chance of a live birth are likely multifactorial. Women may be inadequately informed about their chances—indeed many reported they wished they had been given more realistic information before they started IVF. But insufficient information may not be the only reason why women overestimate chance of IVF success. A study focusing on women aged 43 to 45, aiming to find out why women with extremely low probability of success continue with IVF, suggested it may be due to incorrect interpretation of statistics or ignoring statistical information to follow their wishes (Miron-Shatz *et al.*, 2021). ‘Comparative optimism’, where most people believe their chances are better than that of people in similar situations, may also contribute to the tendency to overestimate chance of success (Kim *et al.*, 2017). In addition, hope likely plays a role. Hope, while considered helpful for increasing resilience in patients, involves the risk that patients ‘anticipat[e] unrealistic futures’ and this may in turn influence their treatment decisions (Perrotta and Hamper, 2021). The well-documented impact of emotion on an individual’s cognitive process may also affect the ability of women having highly stressful and emotional IVF treatment to appraise complex information about chance of success presented to them (Reading, 1989; Blanchette and Richards, 2010). These factors combined may mean that the amount or quality of information provided potentially does not change the decisions women make regarding whether or not to start/continue with IVF, and that these decisions are instead driven by optimism or hope.

While it is widely established that the chance of success with IVF declines with age and is significantly lower in women in their 40s (Cetin *et al.*, 2010), previous studies have demonstrated that this is poorly understood in the general population (Evans *et al.*, 2019). This study suggests that many women undergoing IVF are not aware of this either as we found that women over the age of 40 were more likely than younger women to overestimate their chance of success. This finding may partly be explained by the percentage comparison value calculation, because, due to their lower chance of success, older women had to be more accurate in their estimation to fall within the 20% percentage comparison value bracket. The way that success rates are displayed on IVF clinic websites, with some clinics displaying their overall success rate, as opposed to age-specific success rates, and media reports of women having ‘miracle babies’ through IVF in their 40s may also contribute to this (Hammarberg *et al.*, 2018). Regardless of the cause, the observed high proportion of women over 40 overestimating their chance is concerning, since almost one quarter of women undergoing IVF in Australia are aged 40 years or older (Newman *et al.*, 2021). It highlights the need for women in this age-group to be made aware that IVF cannot reverse the impact of age on reproductive potential (Wyndham *et al.*, 2012). Women may also benefit from information about the pros and cons of using eggs donated by younger women, which significantly increase the chance of live birth (Hogan *et al.*, 2020).

Several of the themes and sub-themes in Fig. 4 reflect the questions asked earlier in the survey, about risks and side effects, chance of success and the treatment process. However, there were also several new themes that emerged from participants’ responses. Of note are the sub-themes of ‘No guarantee of success’, that it ‘May take a long time’ and ‘Emotional and psychological strain’. The first two indirectly point to a lack of understanding about chance of IVF success and further indicate a desire for information that helps women understand how this might impact their IVF journey. It suggests that women not only want to know what their chance of success is, but to be explicitly told what this means for them practically—that it may take a long time and several cycles to be successful, or that they may not be successful at all. This implies a misconception that IVF is a fail-safe treatment, a theme also explored in a qualitative study by Harrison *et al.* (2022). A solution that the authors proposed was moving from cycle-by-cycle planning to multi-cycle planning, where clinicians could implement opportunities for comprehensive information provision and discussion to assist patients in forming realistic expectations of treatment.

In our survey, a number of women also expressed that they wanted more information about the emotional impact of IVF and felt unprepared for this aspect of the treatment. This emphasizes the importance of a holistic and patient-centred approach to information-provision that acknowledges not only the physical demands of the treatment, but the potential emotional and psychological impacts as well. This echoes findings from a survey by Sousa-Leite *et al.* (2023) where the vast majority of participants, who were people undergoing IVF who had not yet been successful, said they wanted to discuss ‘the possibility of treatment being unsuccessful early on in their treatment’ and ‘the bigger picture of what their treatment entails’. Their recommendation was to implement routine psychosocial care into the fertility treatment process, specifically aimed at informing patients about potential outcomes of IVF treatment and equipping them with strategies to manage the emotional impact of treatment and potential failure. Harrison *et al.* (2022) further suggest that clinicians should temper patients’ optimism through transparent discussion of the potential need for multiple IVF cycles. While the need to provide patients with accurate information about their chance of success is evident, it is also important to recognize the impact that receiving this information can have on patients. If this information leads patients to decide to discontinue treatment, they require considerable support for this complex psychological transition (Boden, 2013).

Participants’ preference to receive information from their doctor or nurse highlights this as a target for improvement in information-provision. However, without further investigation into the factors that contribute to women’s lack of understanding of their chance of success, it is not possible to know if modifying the way information is provided will improve their understanding. In fact, Devroe *et al.* (2022) showed that providing patients with a personalized IVF prognosis only lowers the expectations of patients with a lower-than-average chance of success, and even then, only minimally. Despite this, the authors recommend that patients be offered a calculated chance of success, as it improves patient-centredness. The YourIVFSuccess Estimator (National Perinatal Epidemiological and Statistics Unit (NPESU), 2021), based on Australian data, could be used by clinicians to supplement the information they are already providing, with the hope of giving patients a more realistic idea of their chance of success.

While our study has indicated a preference to receive information from an IVF clinician, a randomized controlled trial in the Netherlands found that using an app to provide IVF patients with information relevant to their stage of treatment improves their satisfaction and knowledge (Timmers *et al.*, 2021). This is a strategy that could potentially be employed to reinforce the information provided in consultations by doctors and nurses.

Overall, women in this study did not feel well-informed about the IVF treatment process, the potential risks or side-effects, the probability of having a baby from IVF, or the probability of having a baby without any treatment. The concern relating to this finding is that women may be starting IVF treatment with an unrealistic idea of how likely they are to have a baby, which in turn may limit their ability to weigh up the costs and risks of treatment against chance of success.

### Limitations

As the questionnaire asked women to think back to the understanding they had when they first started IVF, the knowledge and understanding they had gained since they first started may have influenced their responses. We also did not evaluate the trends across the study period and did not compare women who had completed their treatment in 2018 with women who were still having treatment in 2021. Although the study period is relatively short, it is possible that the time since the start of the treatment may have affected recall. These factors combined mean that the responses may not be a completely accurate reflection of participants’ understanding at the time of commencement of treatment. However, this limitation does reflect the reality of ongoing decision-making for patients undergoing in fertility treatment. The bulk of the information is provided at the start of treatment, and recall difficulties may therefore impact patient’s decision-making as time passes.

Volunteer bias may also be present, as the women who were motivated to complete the survey and share their experiences about IVF may not be representative of all women undergoing IVF. It is not possible to know if respondents had a more positive or more negative experience of IVF treatment than non-respondents, or how this might have influenced the findings.

There is inherent imprecision in the way women’s understanding of their chance of success was estimated. The SART calculator is based on a limited number of factors and does not take into account the nuances of individual women’s unique circumstances. For example, it does not include the age of the male partner, which has been shown to impact IVF success rates (Humm and Sakkas, 2013). The calculation also relies on the accuracy of the information provided by the participant, particularly for cause of infertility, and as survey responses were anonymous, this information could not be verified.

While there is now a calculator available based on Australian data (YourIVFSuccess Estimator (National Perinatal Epidemiological and Statistics Unit (NPESU), 2021), this had not been released at the time of this survey, and therefore the SART Calculator was used, which is based on data from the USA. Because success rates are higher in the US overall, due to higher rates of multiple embryo transfer (Meczekalski *et al.*, 2020), it means that our study may be underestimating the degree to which women overestimated their chance.

### Future directions

A prospective study asking women who are about to start IVF about their understanding of their chance of success and gathering data about IVF success and time to pregnancy would be useful to validate the findings from our study. While our findings suggest that good explanations from doctors and nurses about personal chance of success help women gain a more realistic expectation of IVF, they also indicate there is room for improvement. However, in order to develop solutions to improve women’s understanding, it is necessary to understand how they interpret the statistics provided to them and why they are overestimating their chance of success. If the problem is due to the way information is provided by clinics and clinicians, strategies for the improvement of this process can be developed.

Further investigation into adjuncts for information-provision, such as apps, particularly with regard to their impact on patients’ understanding of their chance of success, may also be valuable.

## Conclusion

This study suggests that women undergoing IVF have inadequate understanding of their chance of a live birth with IVF and that most overestimate their chance. The results indicate that women want to be provided with information that allows them to have a realistic expectation of IVF treatment, particularly in terms of their chance of success. While women’s preferred source of information is a consultation with their doctor, our findings suggest that they are not satisfied with the information provided to them by their doctors. This highlights the need for improvement in information-provision, particularly relating to individual chance of live birth, to ensure realistic expectations and informed decision-making.

## Supplementary Material

dead239_Supplementary_Data

## Data Availability

The data underlying this article will be shared on reasonable request to the corresponding author.
