# Supplementary material for: What do women undergoing in vitro fertilization (IVF) understand about their chance of IVF success?
Source: Hum Reprod. 2023 Nov 17;39(1):130–8. doi: 10.1093/humrep/dead239 (PMC10767958; doi:10.1093/humrep/dead239)
Supplement: dead239_Supplementary_Data [file dead239_supplementary_data.pdf]

IVF and You

You are invited to take part in a survey to help us measure what women having IVF understand about their treatment. It includes questions about the types of fertility treatment you have had and what you feel you understand about certain aspects of the treatment process.
This survey is open to all women who have started IVF treatment in Australia in the past three years (in 2018, 2019 or 2020). IVF treatment refers to either an IVF or ICSI cycle, or the transfer of embryos (either created during an IVF/ICSI cycle or through donor eggs or embryos). We are interested in the views of women who started IVF/ICSI recently, therefore women who started IVF in 2017 or earlier are not eligible to take part.

It takes around 10 minutes to complete and your answers will be anonymous, we do not know who you are.

At the end of the survey there is also an opportunity to enter your details to be contacted and interviewed for another related study.

We would like as many women as possible to complete this survey. If you know other women who have started IVF since 2018 please send them this link (copy and paste it):

For more information about the survey, please read the participant information sheet below.

To begin the survey, please click the arrow below.

Participant information sheet

Q3

Do you live in Australia?

○ Yes
○ No

Skip to END OF SURVEY if No is selected

Q4

Are you:

○ Male
○ Female
○ Other
○ Prefer not to say

Skip to END OF SURVEY if Female is not selected

Q5

What type of fertility treatment have you had?

○ Both IVF (including ICSI) and IUI
○ IUI (Intrauterine insemination) only
○ IVF (including ICSI) only
○ None of the above

Skip to END OF SURVEY if None of the above is selected
Skip to END OF SURVEY if IUI (Intrauterine insemination) only is selected

---

Q6

How old are you now? (years)

Select an option

‘18’ to ’55 or over’

Q7

How old were you when you had your first IVF treatment? (years)

Select an option

‘18’ to ’55 or over’

Q8

How tall are you? (cm)

Select an option

‘145 or shorter’ to ‘185 or taller’

Q9

Approximately what was your weight when you first started fertility treatment? (kg)

Select an option

’40 or less’ to ‘120 or more’

Q10

What is your postcode?

Q11

What is your highest level of education?

- ○ Primary School
- ○ Secondary School
- ○ Certificate (including apprenticeship)
- ○ Diploma
- ○ Bachelor’s Degree
- ○ Postgraduate Qualification (e.g. Masters, phD)
- ○ Other

Q12

Please select the cause of your infertility or reason for fertility treatment. *Tick all that apply*

- ☐ Male factor (including low sperm count)
- ☐ Endometriosis
- ☐ Ovulation disorders (including Polycystic Ovarian Syndrome)
- ☐ Decline in ovarian activity (including age-related infertility)
- ☐ Uterine factors (including fibroids)
- ☐ Tubal ligation (tying of the tubes)
- ☐ Tubal disease (blocked or damaged fallopian tubes)
- ☐ Unexplained (no reason was found for either partner)
- ☐ Same-sex couple
- ☐ Single
- ☐ Other

Display this question:
If Q5 = Both IVF (including ICSI) and IUI

Q13

What year did you first start IVF/ICSI?

Select an option

‘Before 2018’ to ‘2021’

Skip to END OF SURVEY if Before 2018 is selected

Display this question:
If Q5 = Both IVF (including ICSI) and IUI

Q14

How many cycles of IUI have you started?

Select an option

‘1’ to ’10 or more’

Display this question:
If Q5 = Both IVF (including ICSI) and IUI

Q15

Did you get pregnant and have a baby from IUI?

- ○ Yes
- ○ No
- ○ Became pregnant but pregnancy did not progress

Display this question:
If Q15 = Yes

Q16

How many babies did you have from IUI?

Select an option ⌄

‘1’ to ’10 or more’

The next few questions ask about IVF/ICSI. We define an IVF/ICSI cycle as a stimulated cycle, normally involving an egg pick-up, and possibly the transfer of a fresh embryo. An embryo transfer can therefore be part of an IVF/ICSI cycle or a separate frozen embryo transfer. (For example, consider a woman who has a stimulated IVF cycle, undergoes egg collection and then has one embryo transferred fresh and 3 embryos frozen. She then returns to have one frozen embryo transferred. This would count as one IVF cycle and two embryo transfers.)

Q18

How many stimulated cycles of IVF/ICSI have you had?

Select an option ⌄

‘1’ to ’10 or more’

Q19

How many embryo transfers have you had, including transfer of frozen embryos? Please provide the number of embryo transfer procedures (e.g. if two embryos were put back at the same time then count this as one embryo transfer)

Select an option

‘1’ to ’10 or more’

Display this question:
If Q18 response is greater than 1

Q20

When was your most recent IVF/ICSI cycle? (month and year)

Month

Year

Q21

Did you get pregnant and have a baby from IVF/ICSI?

- ○ Yes
- ○ No
- ○ Became pregnant but pregnancy did not progress

Display this question:
If Q21 = Yes

Q22

How many babies did you have from IVF/ICSI?

Select an option

‘1’ to ’10 or more’

Q23

In your first cycle, which method was used to fertilise the eggs?

- ☐ Standard insemination
- ☐ ICSI insemination (microinjection)
- ☐ Both standard insemination and ICSI
- ☐ Not applicable (e.g. I did not get any eggs)
- ☐ I don’t know or can’t remember

Q24

In your first cycle, which did you use?

- ○ My own eggs
- ○ Donor eggs
- ○ Donor embryos
- ○ Not applicable (e.g. I did not get any eggs)

Q25

Had you given birth before you started fertility treatment (IUI or IVF/ICSI)?

- ○ Yes
- ○ No

Display this question:
If Q25 = Yes

Q26

How many full-term births (after 37 weeks) did you have prior to starting treatment?

[Select an option ▾]

‘1’ to ’10 or more’

For the next set of questions, we ask you to think back to what you thought when you were about to undergo your first IVF/ICSI cycle.

Q28

At the time of your first IVF/ICSI cycle, how would you rate your understanding of YOUR chance of having a baby from IVF?

	No Understanding						Excellent Understanding				I don't know
	0	1	2	3	4	5	6	7	8	9	10
											☐

Q29

At the time of your first IVF/ICSI cycle, roughly what did you think YOUR chance of having a baby was, after <u>one complete IVF/ICSI treatment cycle</u> (including transfer of all embryos)?

	0	10	20	30	40	50	60	70	80	90	100	I don't know
Percentage chance												☐

Q30

Before starting your first IVF/ICSI cycle, roughly what did you think YOUR chance of having a baby was in a 12-month period with <u>no treatment</u> (i.e. through natural conception/trying at home)?

	0	10	20	30	40	50	60	70	80	90	100	I don't know
Percentage chance												☐

Q31

Before your first IVF/ICSI cycle, which of the following alternative options did your doctor (GP or fertility specialist) or nurse discuss with you? *Tick all that apply*

- ☐ IUI (Intrauterine Insemination)
- ☐ Natural conception (trying at home)
- ☐ Ovulation Induction (using injections or tablets)
- ☐ Hormonal therapies (e.g. to promote ovulation, such as Clomid or Letrozole)
- ☐ Surgery (e.g. to unblock tubes, remove polyps/fibroids or reverse sterilisation in both men and women)
- ☐ Complementary and alternative therapies (e.g. Chinese herbal medicine, acupuncture, etc.)
- ☐ Weight loss and lifestyle change (e.g. with seeing a dietician) and exercise for you and/or your partner
- ☐ Stopping smoking or vaping or use of illicit drugs for you and/or your partner
- ☐ Limiting alcohol intake
- ☐ Other

  ____

- ☐ None of these were options in my case
- ☐ None of these were mentioned

Q32

Which of the following alternative options did you try before you had IVF/ICSI?

- ☐ Natural conception (trying at home)
- ☐ Ovulation Induction (using injections or tablets)
- ☐ Hormonal therapies (e.g. to promote ovulation, such as Clomid or Letrozole)
- ☐ Surgery (e.g. to unblock tubes, remove polyps/fibroids or reverse sterilisation in both men and women)
- ☐ Complementary and alternative therapies (e.g. Chinese herbal medicine, acupuncture, etc.)
- ☐ Weight loss and lifestyle change (e.g. with seeing a dietician) and exercise for you and/or your partner
- ☐ Stopping smoking or vaping or use of illicit drugs for you and/or your partner
- ☐ Limiting alcohol intake
- ☐ Other

  ____

- ☐ I did not try any alternative options

At the time of your first IVF/ICSI cycle, what did you think were the risks involved in your treatment?

Q34

List any possible risks to yourself that you were aware of.

Q35

List any potential risks that you can think of to the embryo, pregnancy or baby

Q36

How much in total have you spent on IVF/ICSI treatment so far? Please provide an approximate of the out-of-pocket costs to you (after being reimbursed by Medicare). Include all extra costs such as medications and additional procedures.

An approximate cost is fine.

Select an option

'Less than $5000' to 'More than $100,000' in $5000 increments

Q37

How did this compare to how much you were expecting to spend?

- ○ Much more than I expected
- ○ A bit more than I expected
- ○ About what I expected
- ○ A bit less than I expected
- ○ Much less than I expected
- ○ I don't know

The following questions relate to how well informed you felt about the process when you first started IVF/ICSI

Q39

How well did your doctor or nurse explain the treatment process to you?

Not well at all	Slightly well	Moderately well	Very well	Extremely well

0 1 2 3 4 5 6 7 8 9 10

Q40

How well did your doctor or nurse explain the potential risks or side effects to you?

Not well at all	Slightly well	Moderately well	Very well	Extremely well

0 1 2 3 4 5 6 7 8 9 10

Q41

How well did your doctor or nurse explain your probability of having a baby from IVF/ICSI?

Not well at all	Slightly well	Moderately well	Very well	Extremely well

0 1 2 3 4 5 6 7 8 9 10

Q42

How well did your doctor or nurse explain your probability of having a baby without any treatment?

Not well at all		Slightly well		Moderately well		Very well		Extremely well	

0 1 2 3 4 5 6 7 8 9 10

Q43

Is there anything you were not told before starting IVF/ICSI treatment that you wish you had been told?

Q44

Overall, how well informed did you feel about your IVF/ICSI treatment before you started?

Not well at all		Slightly well		Moderately well		Very well		Extremely well	

0 1 2 3 4 5 6 7 8 9 10

Q45

How much of your decision to proceed with the IVF/ICSI treatment you had was your decision vs. your doctor's decision?

Your decision — Your doctor's decision — I don't know

0 10 20 30 40 50 60 70 80 90 100

Q46

What is your preferred way(s) of getting information about fertility or fertility treatment?

- ☐ Consultation with doctor
- ☐ Consultation with nurse
- ☐ Videos
- ☐ Written information, e.g. pamphlet
- ☐ Internet searching
- ☐ Journal or research articles
- ☐ Social media
- ☐ Talking to friends/family
- ☐ Books
- ☐ Other

Q47

Do you consent for your individual de-identified (anonymous) response to be shared with other researchers outside the research team?

- ○ Yes
- ○ No

Q48

Would you be interested in being contacted for an interview for a related study about your experiences with fertility treatment? If so, please click on this link and submit your email address or contact number (this is a separate link so that your details are not linked to your survey response in any way).

https://monash.az1.qualtrics.com/jfe/form/SV_dbbSqeYUvZMThKS
